# Foraging Experiences Durably Modulate Honey Bees’ Sucrose Responsiveness and Antennal Lobe Biogenic Amine Levels

**DOI:** 10.1038/s41598-019-41624-0

**Published:** 2019-04-01

**Authors:** Abby Basya Finkelstein, Colin S. Brent, Martin Giurfa, Gro V. Amdam

**Affiliations:** 1 School of Life Sciences, Arizona State University, Tempe, USA; 2 United States Department of Agriculture, Arid Land Agricultural Research Center, Maricopa, USA; 3 Research Centre on Animal Cognition, Center for Integrative Biology, CNRS, University of Toulouse, 118 route de Narbonne, F-31062 Toulouse, Cedex 09 France; 4 Faculty of Environmental Sciences and Natural Resource Management, Norwegian University of Life Sciences, Aas, Norway

## Abstract

Foraging exposes organisms to rewarding and aversive events, providing a selective advantage for maximizing the former while minimizing the latter. Honey bees (*Apis mellifera)* associate environmental stimuli with appetitive or aversive experiences, forming preferences for scents, locations, and visual cues. Preference formation is influenced by inter-individual variation in sensitivity to rewarding and aversive stimuli, which can be modulated by pharmacological manipulation of biogenic amines. We propose that foraging experiences act on biogenic amine pathways to induce enduring changes to stimulus responsiveness. To simulate varied foraging conditions, freely-moving bees were housed in cages where feeders offered combinations of sucrose solution, floral scents, and aversive electric shock. Transient effects were excluded by providing bees with neutral conditions for three days prior to all subsequent assays. Sucrose responsiveness was reduced in bees that had foraged for scented rather than unscented sucrose under benign conditions. This was not the case under aversive foraging conditions, suggesting an adaptive tuning process which maximizes preference for high quality, non-aversive floral sites. Foraging conditions also influenced antennal lobe octopamine and serotonin, neuromodulators involved in stimulus responsiveness and foraging site evaluation. Our results suggest that individuals’ foraging experiences durably modify neurochemistry and shape future foraging behaviour.

## Introduction

Foraging for resources exposes animals to predation and conspecific competition, leading to learned responses to specific locations, smells, visual cues, and other relevant stimuli^1,2^. Individuals can diverge not only in their responses to food-associated stimuli, but in their response thresholds for the nutrients they seek^3^. The mechanisms underlying this inter-individual variation are not well understood. Response thresholds to sucrose, a key nutrient for many species, vary between individual rats^4^, humans^5^, and social insects^6–8^. Research has focused on the genetic correlates of such variability^9–11^, or on the short-term effects wrought by hormonal changes^12,13^, hunger^14–16^, or stress^17–19^. However, there has been little exploration of the long-term effects of food-related experiences on the sucrose response threshold.

The sucrose responsiveness of honey bee (*Apis mellifera*) foragers varies between individuals and causally affects appetitive learning ability^20^. The appetitive response threshold of a honey bee can be quantified using the Proboscis Extension Response (PER) protocol, in which a harness-restrained bee extends its proboscis when the concentration of a droplet of sucrose touched to their antennae is high enough to elicit a feeding reaction^21^. Response thresholds in honey bees are influenced by genotype^16^ and the expression of cyclic guanosine monophosphate (cGMP)-dependent protein kinase (PKG)^22^. Nectar foragers are less responsive to lower sucrose concentrations than pollen foragers^21^, but the overall responsiveness of each group changes throughout the foraging season, particularly in nectar foragers^6^. Additionally, response thresholds can be modulated in the short-term by recent experience: the consumption of scented sucrose acutely increases sucrose responsiveness^23^ while satiation^24^, stress^18^, and the taste of highly concentrated sucrose^16^ have the opposite suppressive effect.

Aversive responsiveness has also been studied in the honey bee using the Sting Extension Response (SER), a defensive response produced by individual bees when facing a noxious, aversive stimulus^25^. The response can be elicited in the laboratory by harnessing a bee between two metal plates and delivering a mild electric shock, which triggers the SER^25–27^. Shock responsiveness is then quantified by exposing the bee to a series of increasing voltages^28^. Aversive thresholds are influenced by caste^28^ and by the exposure to pheromones^29^. Typically, foragers are more responsive to shocks of lower voltages than guards, which respond mostly at higher voltages^28^.

Pharmacological manipulations of biogenic amine circuits have revealed a role in the regulation of appetitive and aversive responsiveness^30,31^. Honey bee sucrose responsiveness is increased by injection or ingestion of octopamine (OA), or its precursor tyramine (TA)^32^. The opposite effect can be induced by injection of dopamine (DA) into the thorax, or by either injection or ingestion of the dopamine receptor agonist 2-amino-6,7-dihydroxy-1,2,3,4-tetrahydronaphthalene (ADTN)^32^. Drugs that antagonize dopamine *D1* and *D2* receptors or serotonin (5-HT) *5-Ht2a* and *5-Ht2b* receptors elevate electric shock responsiveness^33^. It has not been demonstrated that naturally occurring variation in individual responsiveness to sucrose or electric shock correlates with differences in biogenic amine pathways, but adult honey bees typically progress through a series of roles as they mature and age, and some behavioural changes correlate with, and can be induced by, alterations in biogenic amine signaling^34–39^. Octopamine is one of the major neurotransmitters implicated in the transition from working inside the nest to external foraging, and modulates other behaviours such as hygienic maintenance of the hive^40^. Higher levels of OA in the antennal lobes are associated with foraging behaviour and injection can induce an early transition to foraging^34^. Likewise, *OA1* receptors are upregulated in foragers’ antennal lobes and subesophageal zones compared to same-aged honey bees working inside the nest^36^. Furthermore, octopamine mediates the reinforcing properties of sucrose in associative learning protocols in the bee so that octopamine injections act as efficient reward-like stimuli supporting odor learning^41,42^. DA mediates reinforcement signaling in aversive learning of electric shock^31^ and odors associated with aversive taste^43^, but its role in appetitive learning is less clear^44,45^. In this framework, DA signaling would directly substitute for aversive unconditioned stimulus (US), although it may also mediate forms of attention towards this kind of stimulation^33^. The role of 5-HT is less clear in honey bee associative learning as it does not seem to signal the presence of particular forms of reinforcement. Nevertheless, it modulates feeding^46^ and the avoidance of odours associated with ingested toxins^43^, olfactory PER conditioning and retention^30,47,48^, aversive shock responsiveness^33^, and defensive responses to alarm pheromones^49^. 5-HT is also a major neurotransmitter of the bee’s visual system and participates in different forms of visual processing^30,50–52^.

The process of foraging provides individuals with a variety of potential experiences such as encounters with plant olfactory bouquets and nectars, aversive con-specific competition^53^, predation from arthropods and birds^54,55^, as well as noxious secondary compounds secreted by plants^56^. We propose that such diverse foraging experiences induce sustained inter-individual differences in sucrose responsiveness through changes in biogenic amine pathways, and these physiological responses continue to shape individual behaviour after acute effects have subsided.

**Figure 1:**
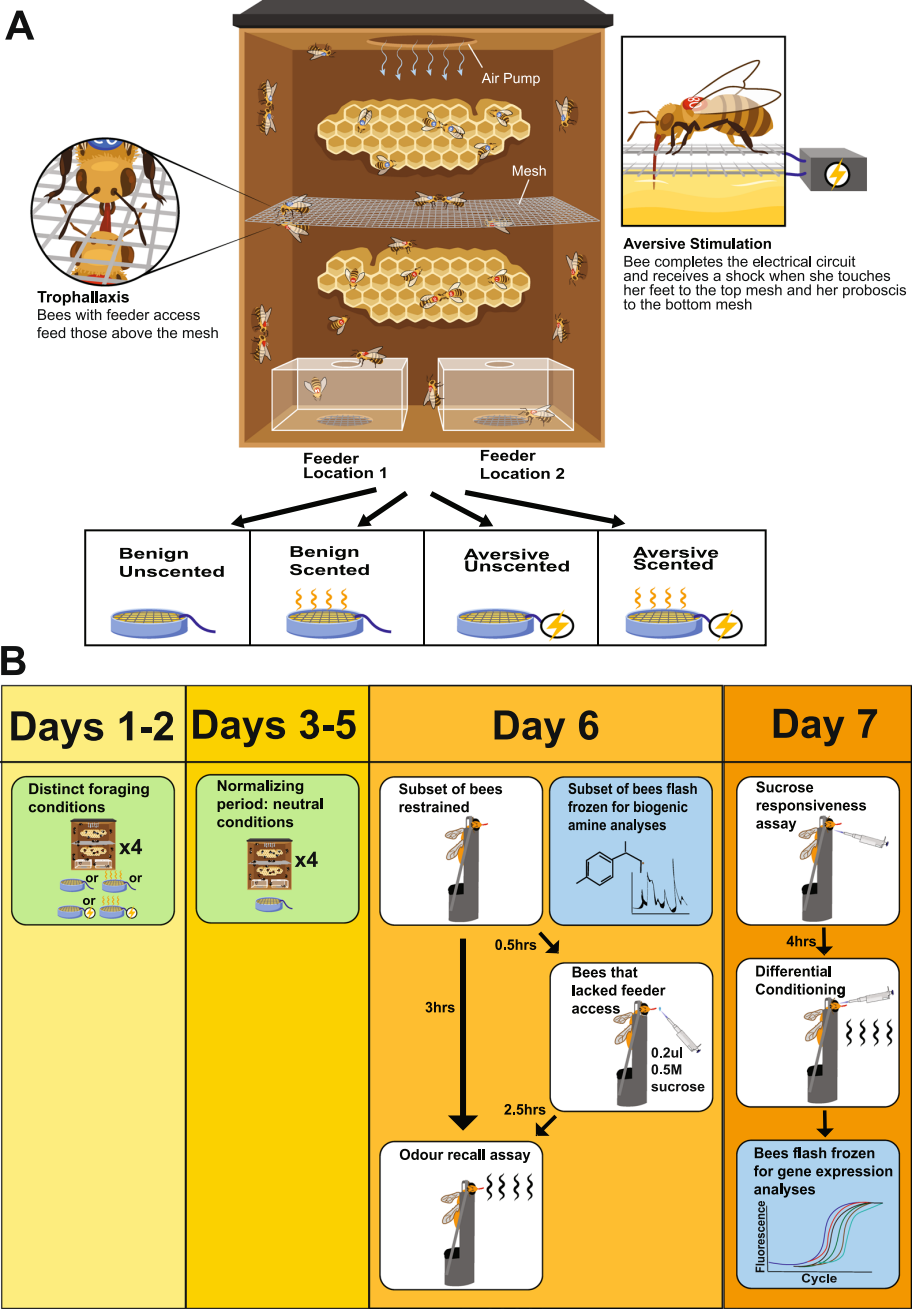
Experimental paradigm. A new experimental paradigm provides free ranging honey bees with feeders paired with electric shock and/or odour. (**A**) Each replicate of the study included four separate cages, each offering different foraging conditions: scented sucrose, unscented sucrose, scented sucrose with electric shock, unscented sucrose with electric shock. The position of food was switched between feeder locations 1 and 2 to avoid spatial associations. Cage dimensions were 14.12 cm wide, 12.85 cm long, and 3.49 cm deep. (**B**) Following differential foraging conditions and a neutralizing buffer period, bees were used for behavioural and molecular assays. Green boxes indicate when bees were caged, white boxes indicate when bees were restrained in harnesses for behavioural assays, and blue boxes indicate when bees were flash frozen for molecular analyses. (**A**) graphic illustration courtesy of Sabine Deviche and the Arizona State University Vislab. © 2018 Arizona State University. Used with permission.

To elucidate the long-term effects of foraging experience, we simulated various foraging conditions using cages in which free-roaming bees could collect food from feeders offering sucrose paired with floral scents and/or electric shock (Fig. 1). Electric shock is the most commonly used form of aversive stimulation in the laboratory for honey bees as well as other model organisms^38,57^. A group of bees fed solely through social feeding, i.e. trophallaxis, was included in each cage to reveal downstream effects of foraging conditions on non-foraging bees. We predicted that bees that had experienced different feeder conditions would exhibit divergent sucrose responsiveness, antennal lobe biogenic amine titers, and receptor expression in the subesophageal zone and antennal lobes, the same regions implicated in a worker’s transition from nurse to forager behaviours.

## Results

### Long-term Recall

**Figure 2:**
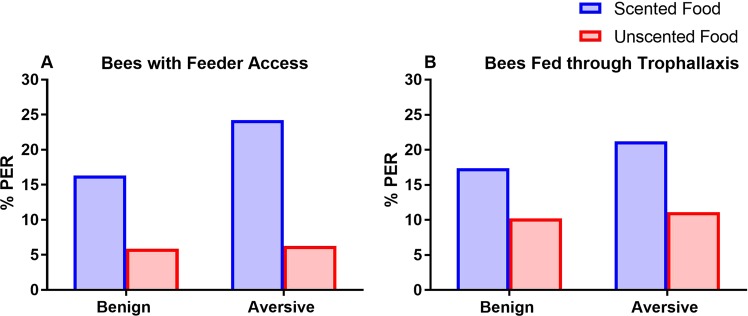
Long-term recall of scented sucrose. Bar graphs show the percentage of bees exhibiting PER (% PER) in response to the floral scent they previously experienced in cages compared to % PER of bees that had not previously experienced the scent. (**A**) Bees with feeder access to scented sucrose (blue) display significantly higher % PER than bees exposed to unscented sucrose (red), regardless of aversive foraging conditions. (**B**) Bees fed scented sucrose (blue) through trophallaxis did not display significantly higher % PER than control bees fed unscented sucrose (red). This data was also used in Fig. 6 of^61^ to address the impact of social information transfer on foraging preferences.

After subjecting bees to different feeding treatments within a cage, we evaluated whether odorants experienced in such treatments would be recalled three days later. Bees were harnessed individually and exposed to odorants to determine whether they elicited PER, revealing their prior association with food. The specific scent previously experienced in cages (linalool vs phenyacetaldehyde) did not influence recall (Logistic Generalized Estimating Equations, bees with feeder access – Scent Type: β1 Wald χ^2^ = 1.22, p = 0.269, n = 82, bees without feeder access – Scent Type: β1 Wald χ^2^ = 1.1, p = 0.295, n = 79), so in both cases we pooled bees that had experienced the two different floral scents. Bees that previously had access to feeders exhibited recall, as was evident from a higher PER in response to the scent paired with sucrose, which occurred at double the rate for scent that was not paired with food (Fig. 2A: Logistic Generalized Estimating Equations, β1 Wald χ^2^ = 7.14, p = 0.0075). There was no significant impact of electric shock at the feeder (Fig. 2A: β1 Wald χ^2^ = 0.602, p = 0.438). Bees that previously fed on scented sucrose solely through trophallaxis did not exhibit long-term recall, exhibiting similar PER rates for scent that had been paired with sucrose and for scent that had not been paired with sucrose (β1 Wald χ^2^ = 2.57, p = 0.109). Aversive conditions experienced by foragers had no downstream effect on the recall of recipients of trophallactic exchanges of scented food (β1 Wald χ^2^ = 0.173, p = 0.678). Sample sizes for bees with feeder access is as follows – scented benign: 49, scented aversive: 33, unscented benign: 51, unscented aversive: 48. Sample sizes for bees lacking feeder access is as follows – scented benign: 46, scented aversive: 33, unscented benign: 49, unscented aversive: 54. Overall, the observed levels of PER to appetitively associated scent were generally low, possibly as a result of the stress induced by a prolonged time spent in cages and being chilled on ice twice in a five-day period^58,59^. Previous studies examining memories formed by caged bees tested recall after <24 hr^23,60^ rather than the 72 hrs used here.

### Sucrose responsiveness

**Figure 3:**
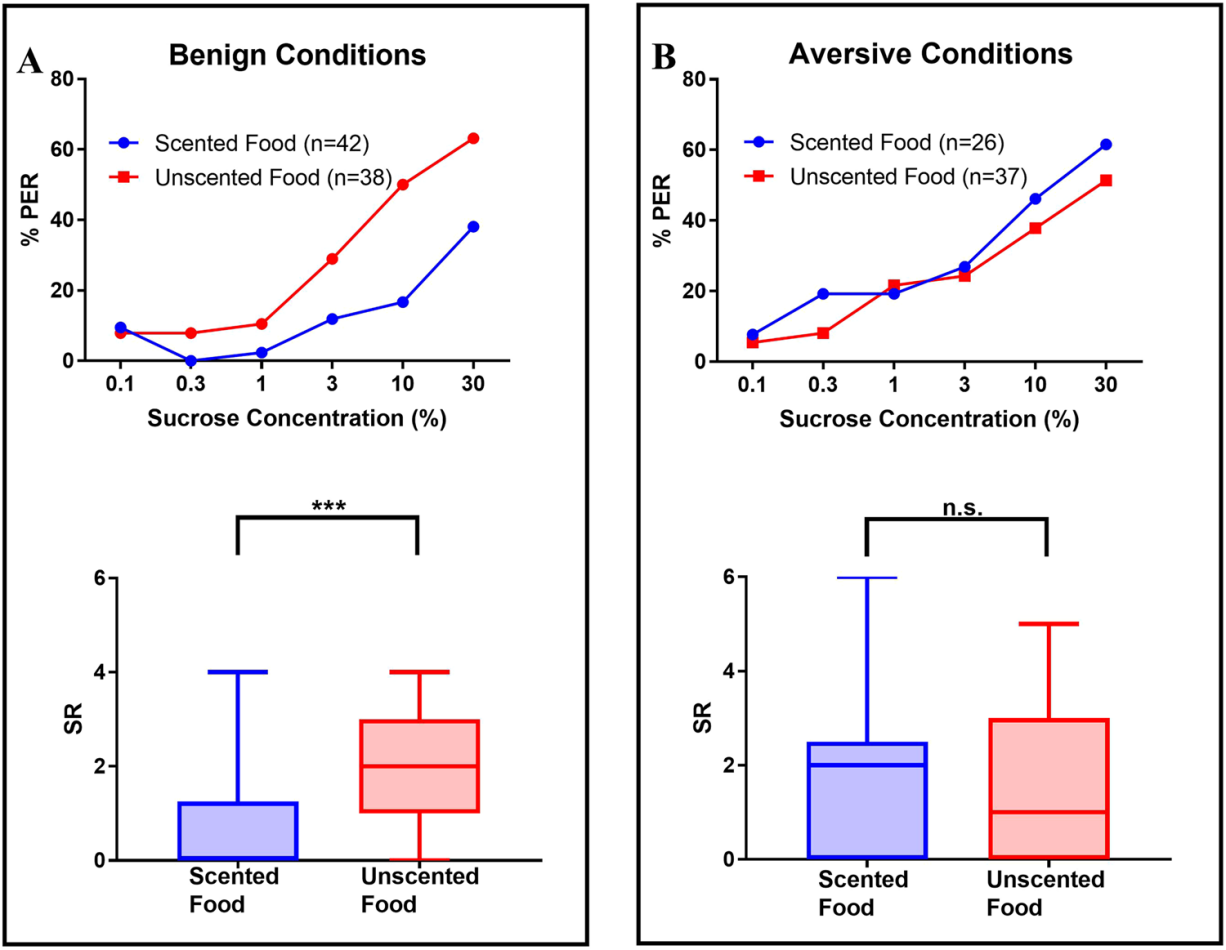
Long-term effect of scented sucrose on bees with feeder access. Curves show the percentage of bees responding with a proboscis extension response (% PER) to increasing concentrations of sucrose. Boxplots below show sucrose responsiveness scores (SR); boxes represent 25^th^–75^th^ percentiles, solid lines indicate medians, and bars show minima and maxima. (**A**) In benign foraging conditions, sucrose responsiveness score (SR) in bees with feeder access is reduced by scented food (blue) relative to unscented food (red). (**B**) Aversive foraging conditions eliminate the effect of scent on SR. This figure is based on a subset of the data used in Fig. 3 of^61^ to determine differences between foragers and non-foragers.

**Figure 4:**
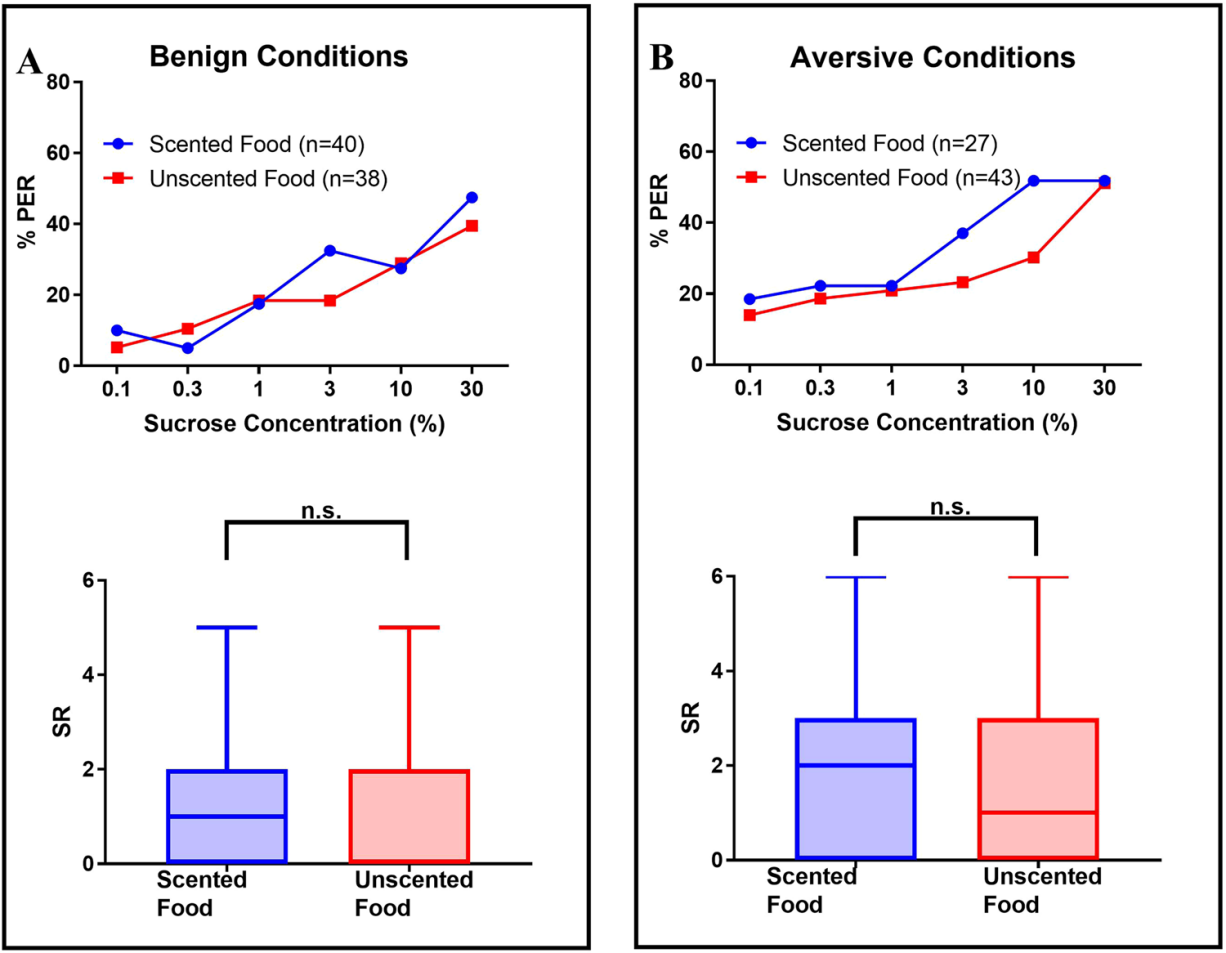
Long-term effect of scented food on bees fed solely through trophallaxis. Curves show the percentage of bees responding with a proboscis extension response (% PER) to increasing concentrations of sucrose. Boxplots below show sucrose responsiveness scores (SR); boxes represent 25^th^–75^th^ percentiles, solid lines indicate medians, and bars show minima and maxima. In both benign (**A**) and aversive (**B**) foraging conditions, there is no effect of scented food on the SR of bees fed through trophallaxis. This figure is based on a subset of the data used in Fig. 3 of^61^ to determine differences between foragers and non-foragers.

To characterize long-term effects of different foraging experiences on sucrose responsiveness (SR), we performed SR assays on harnessed bees one day after they underwent long-term recall tests (four days after the cessation of differential treatment). For bees with feeder access, the presence of scent dissolved in sucrose in benign foraging conditions reduced subsequent sucrose responsiveness compared to bees that had experienced unscented food (Fig. 3A, upper panel). (Fig. 3A, lower panel; Mann Whitney U test: n_no scent_ = 38 and n_scent_ = 42, U = 468, p = 0.0008). Aversive foraging conditions eliminate the effect of scent on subsequent sucrose responsiveness; there is no difference in sucrose responsiveness between bees that had access to scented vs unscented food when paired with electric shock (Fig. 3B, upper panel). Bees that had access to scented vs unscented sucrose exhibited the same SRs (Fig. 3B, lower panel; n_no scent_ = 37, n_scent_ = 26, U = 428, p = 0.4480) (Fig. 3A,B). On the other hand, bees that were fed solely through trophallaxis did not exhibit differences in sucrose responsiveness due to presence of scent, irrespective of the appetitive or the aversive conditions experienced by the food donors (Fig. 4; benign conditions, n_no scent_ = 38, n_scent_ = 40, U = 689, p = 0.451; aversive conditions, n_no scent_ = 43, n_scent_ = 27, U = 501, p = 0.318). Dataset also used in^61^.

### Impact of long-term recall on sucrose responsiveness

We hypothesized that the reduction in SR by scented sucrose in benign conditions was mediated by the process of forming memories associating sucrose with scent. If this were the case, one would expect bees that successfully retrieved long-term memories to exhibit lower SRs than bees that experienced scented sucrose but failed the long-term recall test. However, we found no difference in SR between bees that succeeded and failed the long-term recall test after experiencing scented sucrose in benign foraging conditions (Mann Whitney U Test: n_no recall_ = 30, n_recall_ = 6, U = 86, p = 0.876), indicating that the effect of scent on subsequent sucrose responsiveness is not mediated through the process of forming long term memory. Dataset also used in^61^.

### Biogenic amine titers

**Figure 5:**
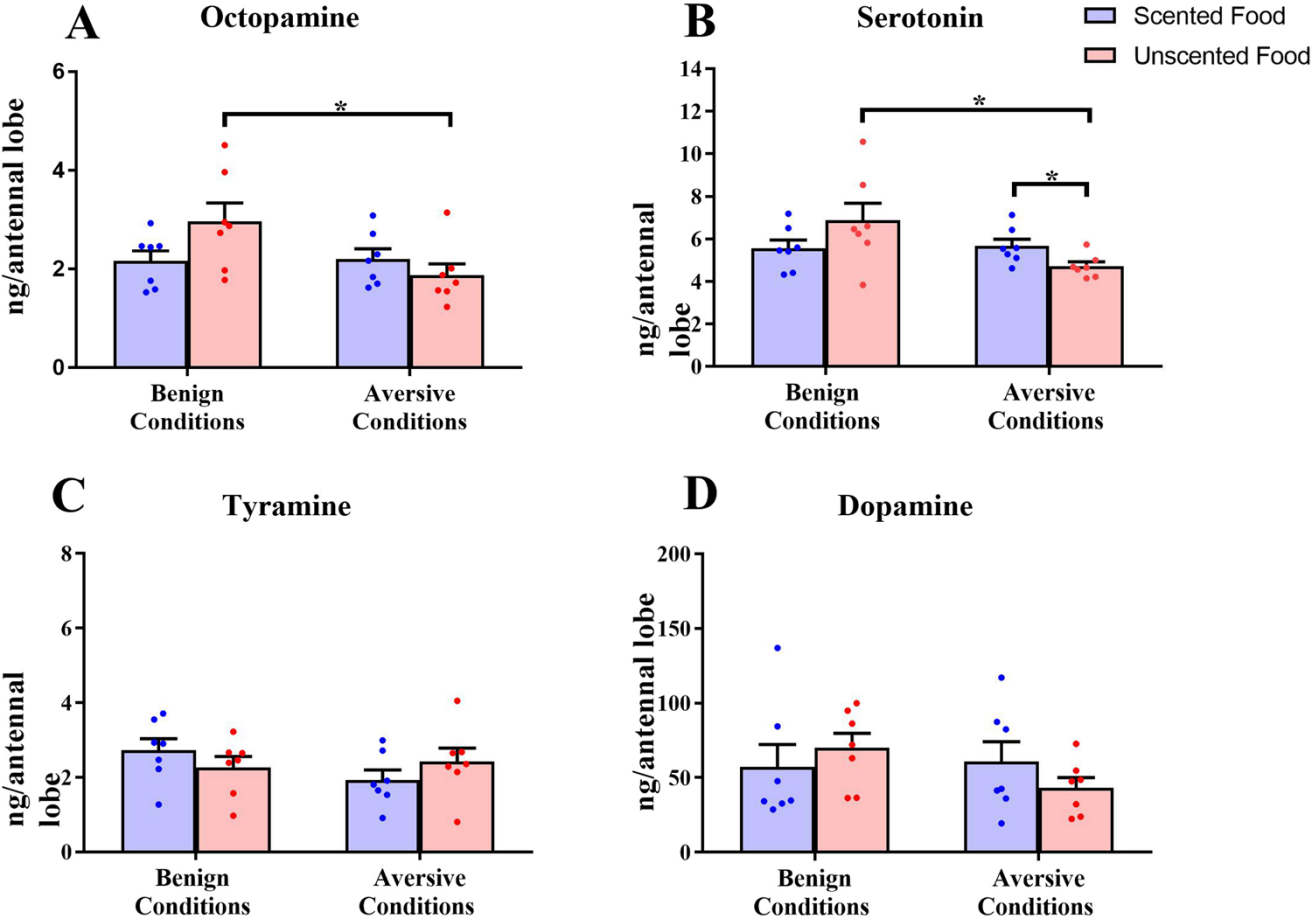
Antennal lobe biogenic amine concentrations in bees with feeder access, 2 days after differential foraging conditions. Bars show mean concentrations, error bars show s.e.m., circles show all raw data points. For unscented food (red), aversive conditions reduce antennal lobe serotonin and octopamine concentrations. In aversive conditions, scented food (blue) elevates serotonin concentrations.

Biogenic amine pathways are a likely substrate for lasting behavioural change. To explore the influence of differential foraging experiences on subsequent biogenic amine signalling, biogenic amine levels were quantified in the brains of bees removed directly from the cages after the 3 day normalizing period. When food was unscented, aversive foraging conditions reduced concentrations of octopamine (OA) (Fig. 5A; Mann Whitney U Test, p = 0.0379, U = 8, n = 7,7) and serotonin (5-HT) (Fig. 5B; p = 0.0262, U = 7, n = 7,7) in antennal lobes relative to benign conditions. Under aversive foraging conditions, 5-HT increased when food was scented relative to unscented (Fig. 5B; p = 0.0379, U = 8, n = 7,7). Tyramine (TA) did not vary with odour (Fig. 5C; p = 0.1282, U = 12, n = 7,7) or foraging conditions (p = 0.3829, U = 17, n = 7,7). Similarly, DA was invariant regardless of odour (Fig. 5D; p = 0.0728, U = 10, n = 7,7) or conditions (p = 0.5350, U = 19, n = 7,7).

### Octopamine receptor expression

We next determined if differences in sucrose responsiveness in the behavioural assays correlated with differences in biogenic amine receptor gene expression in the honey bee brain. We focused our analysis on bees with feeder access in benign conditions as they exhibited significant differences in sucrose responsiveness depending on the presence of odorant in the food. Due to the previously described relationship between octopamine and SR, we examined expression levels of octopamine receptor *AmOA1* as it is expressed differentially based on behavioural role rather than age in the subesophageal zone and antennal lobes of freely flying bees^36^.

**Figure 6:**
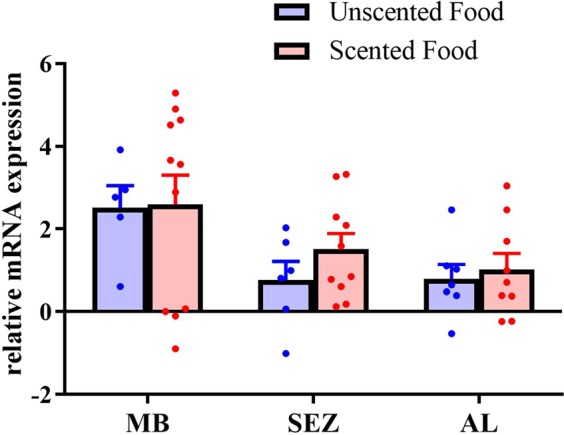
*AmOA1* expression levels in bees with feeder access after exposure to scented vs non-scented food in benign foraging conditions. Bars show mean concentrations, error bars show s.e.m., circles show all raw data points. There were no differences in expression in any of the three tested neuropiles for bees with feeder access. This data was part of a larger dataset used to determine differences between foragers and non-foragers in Fig. 4 of^61^.

The effect of scent on sucrose responsiveness was not related to a difference in *AmOA1* expression in antennal lobes (Fig. 6 AL: Student’s t-test, n_non-scented_ = 7, n_scented_ = 9, t = 0.422, df = 14, p = 0.6794), subesophageal zone (SEZ: n_non-scented_ = 10, n_scented_ = 6, t = −1.246, df = 14, p = 0.2333), or mushroom bodies (MB: n_non-scented_ = 5, n_scented_ = 11, t = 0.07599, df = 14, p = 0.9405). Subset of this dataset also used in^61^.

## Discussion

Our study found lasting effects of differential foraging experiences on sucrose responsiveness and biogenic amine titers of the antennal lobes. Honey bees with access to benign feeders containing scented sucrose solution exhibit a long-term reduction in sucrose responsiveness relative to bees with access to unscented sucrose solution (Fig. 3), indicating heightened foraging selectivity. This effect of scent does not extend to bees fed through trophallaxis (Fig. 4), suggesting that incoming food stimuli have a limited long-term effect on pre-foragers. Aversive foraging conditions, simulated via electric shock at feeders, suppress the effect of scented sucrose on sucrose responsiveness. Foraging conditions also impact antennal lobe levels of octopamine and serotonin (Fig. 5), biogenic amines that regulate appetitive and aversive stimulus responsiveness^32,33,49^. These results suggest that dissimilar foraging experiences in the field could enduringly contribute to inter-individual differences in stimulus responsiveness.

Scented sucrose lastingly modulates the sucrose responsiveness of only the caged bees with direct feeder access, suggesting that this effect is facilitated by the physiological modifications that accompany the transition to foraging behaviour or by the behavioural context of foraging. In the latter case, the encoding and impact of appetitive scented experiences may vary depending on whether they are acquired directly through foraging or via a social exchange. In both cases, bees can learn odour-sugar associations as it has been shown that bees can respond with PER one day after experiencing the odour during trophallactic food exchanges^62,63^. However, it is possible that only the information acquired while foraging is salient enough to induce long-term changes in sucrose responsiveness. In the field, this differential impact could translate to long-term effects on foragers but not pre-foraging bees inside the colony. Long-term reductions of sucrose responsiveness in the case of foragers collecting high quality scented sucrose in non-aversive conditions could adaptively maintain selectivity for food sources, promoting returns to the same source as long as quality persists rather than settling for sources of lesser quality. In contrast, the foraging landscape is likely to have changed by the time the pre-foragers begin to make their own foraging decisions so they have no selective advantage in long-term retention of modulated sucrose responsiveness. Pre-foragers are still primed to experience short-term modulation, as they increase their sucrose responsiveness when assayed 24 hrs after consuming scented sucrose in both cages and outdoor colonies^23^. The diverging effects of scented sucrose on foraging vs downstream non-foraging bees at different time points provide an intriguing direction for future studies.

Pairing scent with food results in associative learning, giving rise to the possibility that learning alters not only an individual’s responsiveness to the conditioned stimulus (scent) but also to the unconditioned stimulus (sucrose). However, there is no difference in sucrose responsiveness between bees that display or do not display long-term recall, a metric of the consolidated long-term memory that results from learning (see results). Moreover, aversive foraging conditions eliminate the effect of scent on sucrose responsiveness (Fig. 3B), but do not significantly impact associative learning as quantified by long term recall (Fig. 2). It is still possible that learning plays a role; short-term learning, which we did not assay, could contribute to the long-term effect of scent on sucrose responsiveness. Note that the overall PER% is low compared to other studies in which free-flying or caged bees were restrained in harnesses for long-term recall^23,60^, likely due to a prolonged period of time in caged conditions between learning and recall, which was three days rather than the usual 24hrs.

We speculate that there are two, potentially interacting, neural mechanisms that could give rise to the long-term change in sucrose responsiveness of a forager. The first possibility is that the change in responsiveness occurs at the level of antennal peripheral sucrose receptors, potentially through biogenic amine modulation as shown for olfactory receptors in several species of moths. In the cabbage moth *Mamestra brassicae* octopamine increases the likelihood of firing while serotonin inhibits firing of pheromone receptors^64^, and in the silkmoth *Bombyx mori* octopamine selectively enhances sensitivity to pheromones^65,66^. To determine whether scented sucrose induces peripheral sensory changes, future studies could quantify biogenic amines and gustatory receptor expression at the level of the antennae and perform single-sensillum recordings of taste sensilla^67^ to compare firing rates. A second possibility refers to central modulation of attention towards appetitive stimuli by experiencing the combination of scent and high quality sucrose. Such experience could direct attention toward sensing olfactory stimuli that could predict high sucrose reward. Prediction and attention are interdependent^68^, and attention amplifies sensory processing of attended features and suppresses processing of irrelevant features^69–71^. In the honey bee, attentional modulation of sucrose responsiveness may occur either in the SEZ or in integrative structures such as the mushroom bodies as the latter have been shown to modulate visual attention in flies^72^ and bees^73^. Although we find no differences in mushroom body expression of octopamine receptor *AmOA1*, other octopaminergic receptors identified in the bee genome could underlie these processes (Fig. 6)^36,74,75^. Furthermore, more targeted techniques may be able to identify changes to specifically olfactory versus gustatory circuitry.

In aversive foraging conditions, scented food induces a sustained elevation in antennal lobe serotonin compared to unscented food (Fig. 5B). A similar effect has been found when bees are exposed to the main alarm pheromone component isoamyl acetate^49^, thus suggesting that serotonin is a key player in the regulation of responses to noxious stimuli. Serotonin antagonists elevate electric shock responsiveness to lower voltages in the honey bees, leaving intact responsiveness to higher voltages^33^. This suggests that elevated serotonin could lead to reduced responsiveness to aversive stimuli of lower intensity^33,38^, which could help focus responses on relevant and harmful stimuli. A depression in aversive responsiveness following the pairing of electric shock with a cue is consistent with preliminary work showing that aversive learning leads to a reduction in shock responsiveness to intermediate voltages (Finkelstein *et al*. unpublished; Tedjakumula *et al*., personal communication). The ecological equivalent of scented sucrose paired with electric shock is a hazard at a recognizable food source. Examples of such situations include predators that specialize in hunting at specific types of plants^54,55^, food patches located close to predator nests^76^, or types of plants producing bitter toxins^56^. In such cases, there is an adaptive benefit to avoid high-risk food sites while becoming more accepting of less aversive stimuli at other food sites.

Aversive conditions are not always predicted by a recognizable scent, as they are not always specific to a patch of flowers or type of flower. Predation can provide generalized, unpredictable danger; in some environments, overall predation pressure can vary between years^76^. Conspecific competition at plant sites is also aversive to honey bees^53^; crowding, such as in commercial beekeeping operations, could result in high overall con-specific competition at food sites. Within our paradigm, electric shock at feeders with no associated odour may simulate unpredictable ecological danger. In these unscented and thus unpredictable conditions, we observe reduced antennal lobe OA and 5-HT concentrations in bees that experienced aversive rather than benign foraging conditions (Fig. 5A,B). Reduced 5-HT concentrations could result in increased responsiveness to shock as suggested by pharmacological experiments reporting that 5-HT antagonists elevate responsiveness to electric shock^33^. OA mediates the appetitive, reinforcing properties of sucrose solution in the bee brain^32,41,42,47,77^. The decrease in OA levels could thus reflect the reduction in the appetitive value of a food source that is associated with an aversive reinforcement. Accordingly, ingestion of OA causes bees to be less likely to learn to avoid an area where they have received electric shock^78^. Reducing OA would be expected to have the opposite effect, accelerating aversive learning; future work could test this by training bees in an aversive conditioning task to compare performance between bees with different foraging experiences and OA levels. We propose that the reduction of 5-HT and OA in unpredictably aversive foraging conditions coordinates an increase in aversive responsiveness and learning to accelerate acquisition of aversive associations.

Taken together, our findings demonstrate that foraging experiences durably influence caged honey bees’ sucrose responsiveness, and reveal neurochemical changes that likely mediate additional effects on behaviour. We hope these findings spur future field studies exploring the behavioural consequences of experience-induced changes in biogenic amine levels and sucrose responsiveness.

## Methods

### Bee Collection

Honey bee (*Apis mellifera ligustica*) foragers were collected from hives at the Arizona State University Campus in Tempe, AZ from April-June 2015 in the morning. For each test date, bees were taken from three different hives and mixed together to minimize hive-specific effects. Equal numbers of bees from the 3 different colonies were mixed for each of the different treatments to ensure that any impact of mixing bees from different hives would be the same across conditions. Once bees had been chilled on ice and placed together in the new cage context, they did not appear to exhibit any aggression towards one another. Recent work suggest that interactions are more frequent between prior nestmates vs non-nestmates^79^, but our assays are not dependant on specific networks of interaction. Mature but pre-foraging nest bees were chosen to avoid variation due to aging^80^. We chose bees by lifting the outer honey comb frames, where younger nest-bees are unlikely to be found^81^, out of the hive box, and collecting only those bees that did not fly away when gently touched with soft forceps. Bees were placed in glass vials, and cooled on ice until they stopped moving.

### Cage Paradigm

Once immobilized, each individual was labelled with bee number tags (The Bee Works Queen Marking Kit, Ontario, Canada) on the thorax, and placed inside an experimental cage in which they regained motility and became acclimatized for three hours before experiment initiation. To simulate the dichotomy between the foragers and nest workers in a eusocial insect colony, each cage was divided by mesh to separate bees into two groups: 17 bees with feeder access in the bottom compartment and 13 bees with no feeder access in the top compartment (Fig. 1). The bees in the top compartment were fed via trophallaxis events during which at least one bee’s proboscis is between the mandibles of another bee as liquid food is transferred, accompanied by antennation^82–84^. These two groups also allowed us to separate overall cage effects such as alarm pheromones that diffuse throughout the cage, odor contamination, and the stressful experience of being caged from the specific effects of foraging in particular conditions. Empty honey comb lined the back wall in both compartments. Six replicates were run, each consisting of four cages, differing only in the feeder conditions for the first two days. The cages were exposed to constant fluorescent lighting and were kept at room temperature (21–26 °C). The four locations for cages within a room in our laboratory were kept constant, and the location of each experimental condition was alternated between replicates. In the two boxes simulating aversive foraging conditions, the first two days of treatment consisted of two Kimwipes (Kimberly-Clark Professional, Roswell, Georgia, USA) soaked in 1.5 M sucrose in a feeder that delivered 4.2 V of electric shock to bees that completed the circuit by touching their feet to the top electrical mesh and their proboscises to the bottom electrical mesh (Fig. 1). Piloting with different voltage strengths showed that 4.2 V produced a slight startle response but did not prevent bees from feeding. In one of these boxes, the sucrose was scented (50ul odourant per liter of 1.5 M solution) with floral compounds linalool or phenylacetaldehyde (Sigma Aldrich, St. Louis, MO, USA)^23^. Odour was alternated between replicates, with the same odour was used for each treatment within a replicate, to demonstrate that any effects of odour were not specific to one particular floral compound. The other two boxes received identical treatment but without the electric shock, simulating benign foraging conditions. Two feeder slots at the bottom of each cage allowed food location to be alternated every 6–8 hours to reduce spatial associative pairing^85–87^. After two days of treatment, the feeder slots at the bottom of all boxes were cleaned with a Kimwipe dampened with ethanol to remove traces of sucrose, odourant, or secretions from honey bees. To provide a neutral buffer period between the differential foraging conditions and subsequent assays, all feeder slots were filled with bottle caps containing two Kimwipes soaked with 0.5 M sucrose and replaced to maintain saturation. This low sucrose concentration was used to prevent a floor effect on subsequently assayed sucrose responsiveness, since recent exposure to high concentrations of sucrose decreases sucrose responsiveness^16^.

After three days of *ad libitum* feeding, cages were laid horizontally to allow minimally stressful collection of bees by allowing them to climb up into tubes. We alternated between collecting bees in glass tubes for behavioural assays, and collecting bees in plastic Eppendorf tubes to be flash frozen in liquid nitrogen for biogenic amine analysis. Only bees from the compartment with feeder access were included in the latter group, as the bees without feeder access did not exhibit changes in sucrose responsiveness.

### Biogenic Amine Analysis

Biogenic amine concentrations can change rapidly in bees^88^, especially due to stress^89^, so we chose to analyse the biogenic amine profiles immediately following removal from cages rather than after behavioural assays. Flash frozen bees were stored at −80 °C. Antennal lobes were later dissected from frozen samples. A detection limit of 25 pg precluded the analysis of individual antennal lobes, so they were pooled in groups of five pairs of antennal lobes per sample. High-performance liquid chromatography (HPLC) was used for analysis of biogenic amine content following established protocols^35,90^. Dissected antennal lobes were placed in a 1.5-ml centrifuge tube and homogenized with a pestle in 20 µl of chilled perchloric acid (0.2 M) containing dihydroxybenzylamine (DHBA, 87 pg/µl; Sigma-Aldrich, St. Louis, MO, USA) and synephrine (50 pg/µl; Sigma-Aldrich) as internal standards. Samples were then sonicated for 5 min in a covered ultrasonic bath (Branson 2510, Branson Ultrasonics Corp., Danbury, CT, USA) filled with an ice water slurry. After sonication the samples were allowed to sit in the water bath for an additional 20 min to maximize amine extraction. Samples were spun at 12,000 RCF for 10 min in a refrigerated (4 °C) centrifuge, then kept on ice in a covered container until analysis.

Only six samples were prepared at a time to minimize the delay between removal from the freezer and amine quantification. The biogenic amine content of 10 µl of supernatant was determined on an HPLC system (ESA, Chelmsford, MA, USA) consisting of a Coularray model 5600A with a 4 channel electrochemical detector (Ch1 650 mV, Ch2 = 425 mV, Ch3 = 175 mV, Ch4 = −125mV), a model 582 pump, and a reverse-phase catecholamine HR-80 column. Samples were manually injected (Rheodyne 9125, Rohnert Park, CA, USA) into a 20-μl loop. Mobile phase (flow rate = 0.5 ml min^−1^) consisted of polished water, 15% methanol, 15% acetonitrile, 1.5 mmol l^−1^ sodium dodecyl sulfate, 85 mmol l^−1^ sodium phosphate monobasic, and 5 mmol l^−1^ sodium citrate. Phosphoric acid was used to adjust the buffer pH to 5.6. Results are expressed on a per head basis. The size of resultant peaks was compared to a serial set of external standards (hydrochloride forms of DA, OA, 5-HT, TA; Sigma-Aldrich) run before and after each set of 6 samples to determine the equivalent quantity in picograms.

### Behavioural Assays: Recall, Sucrose Responsiveness, and Differential Conditioning

Bees in glass vials were chilled on ice until immobilized, and then restrained individually in metal harnesses with strips of duct tape (Duck Brand, Avon, OH, US^91^. After a 20 minute rest period, bees from the compartments without feeder access were fed with 2 ul of 0.5 M sucrose from a pipette tip by first touching the antennae to elicit the Proboscis Extension Response (PER); extension of the proboscis beyond an imaginary line between the opened mandibles^91,92^. We did not feed the bees that had access to sucrose, as these bees were already presumed to have fed and our goal was to bring all bees to a similar state of satiation. Due to numerous experiential differences between bees with and without feeder access, comparisons were never made between these two groups. Three hours after harnessing (i.e. 3 days after removal of scents), all bees were tested for retrieval of scent memory by recording PER to the floral scent used in cages and to a novel scent used as a control for the specificity of the memory retrieved^92^. Memories retrieved 3 days after olfactory PER conditioning have been characterized as long-term memories due to their dependence on protein synthesis^45,92,93^. We used 2-octanol or 2-hexanol (Sigma Aldrich, St. Louis, MO, USA) as novel scents due to their prevalent use in studies concerning olfactory memory and perception^92,94,95^. Odour cartridges consisted of a glass 1 cc tuberculin syringe barrel (BD Medical, Franklin Lakes, NJ) and silicon tubing (Cole-Parmer, Vernon Hills, IL) which were used to deliver scented air. 10 μl of pure odour was soaked into a strip of filter paper (Whatman 114, Sigma-Aldrich, St. Louis, MO) pushed into the cartridge. Silicon tubing attached the narrow end of odour cartridges to an automated odour delivery system, so that the wide end stayed approximately 2 cm from bees’ antennae. A DirectLogic 05 programmable logic controller (Automation-Direct, Cumming, GA) coordinated scent delivery by opening a valve (The Lee Co., Westbrook, CT) releasing an airstream (~400 ml/min) over the scented filter paper for 4 seconds. A continuous flow exhaust system ~5 cm behind the bee cleared scent from the testing arena. Each bee was presented once with whichever scent had been used in the cages with scented sucrose (linalool or phenylacetaldehyde^23,63^) and once with novel scent (2-octanol or 2-hexanol) in a pseudo-random order, with more than 10 minutes between trials. Bees’ responses to each scent were recorded as a binary “yes” or “no,” with “yes” indicating that bees exhibited PER during presentation of scent and before presentation of sucrose^91,92^. After the last trial, all bees were given *ad libitum* 0.5 M sucrose until touching the antennae with a droplet of sucrose did not induce PER, indicating satiety.

The following morning, 96 hours after the cessation of differential foraging conditions, sucrose responsiveness was determined by presenting every bee (randomly ordered) with a progressive sequence of 0.1%, 0.3%, 1%, 3%, 10%, 30% sucrose (w/w) interspersed with water stimulations between each trial to prevent sensitization according to standard protocol^16,18,96,97^. Bees that responded to water were excluded from analyses. The interval was always greater than 10 minutes and was the same for bees in all treatment groups within each replicate, as is standard procedure in Recall and Sucrose Responsiveness assays^18,21,97^. Both antennae were touched with a droplet of sucrose or water on a pipette tip, and PER was recorded to produce a Sucrose Responsiveness score (SR) of 0–6, indicating the number of sucrose concentrations to which the individual responded^97,98^.

Four hours after being used in the sucrose responsiveness assay, bees were tested for their ability to learn new appetitive olfactory associations in a discrimination assay. One odour (CS+) predicted reward of 1.5 M sucrose while the other (CS−) did not predict reward. The two odours used included whichever floral scent (linalool or phenyacetaldehyde) and non-floral scent (2-octanol or 2-hexanol) had not been previously used in the long-term recall test. Each trial consisted of a few seconds of acclimatizing to the testing context, followed by a 4 second presentation of the odour stimulus by pushing air through an odour cartridge. Three seconds after odour onset, the CS+ odour was forward-paired with 0.6 μl of 1.5 M sucrose while the CS− odour was not paired with sucrose. Afterward, the bee was left in position for several seconds. Each bee had an inter-trial interval of at least 10 minutes. Approximately 30 minutes following conditioning, we tested short-term retrieval by exposing each bee to a single unreinforced test trial with each odour, alternating the order of odours presented. Unfortunately, high stress levels prevented an accurate demonstration of learning ability. Stress was evidenced by drooping heads, slightly extended proboscises, reduced locomotion inside harnesses, and a lack of proboscis extension response when touched on the antennae with 1.5 M sucrose during the CS+ trials. All but a few bees failed to learn during the conditioning, therefore the resulting data are not presented. The methods for this assay are included in case they affected subsequent gene expression assays.

### Octopamine Receptor Gene Expression Analysis

Bees were flash frozen in liquid nitrogen immediately after behavioural assays. Our intent was to determine expression difference between bees that had exhibited differences in performance on behavioural assays, therefore only bees with feeder access in benign conditions were used. Their mushroom bodies, antennal lobes, and subesophageal zone were dissected into separate reaction tubes for RNA extraction and subsequent gene expression analyses. Total RNA was extracted from each neuropile using a trizol/chloroform protocol (ThermoFisher Scientific, Waltham, MA, USA). DNase treatment was performed with the RNase-Free DNase Set (Qiagen, Hilden, Germany). A NanoDrop™ 2000/2000c Spectrophotometer (ThermoFisher Scientific, Waltham, MA, USA) was used to provide RNA concentrations and purity as characterized by 260/280 ratios ranging from 1.8 to 2. cDNA synthesis was performed in duplicate (technical replicate) for each biological sample, using 100 ng total RNA per reaction, with the Taqman Reverse Transcription Reagents Kit (ThermoFisher Scientific, Waltham, MA, USA).

Using well-established *OA1* primers^36^, we performed quantitative real-time Polymerase Chain Reaction (qrtPCR) using the ABI PRISM® 7000 Sequence Detection System (ThermoFisher Scientific, Waltham, MA, USA) and Quantitech SYBR® Green RT-PCR kit (Qiagen, Caldwell, NJ, MA) to compare expression levels between bees that had foraged for scented vs unscented sucrose. For normalization of the receptor transcripts, we used *elongation factor 1 alpha* as the reference gene, because it is stably expressed in nurse bees and foragers, castes that are known to exhibit different sucrose responsiveness^36^. Three technical replicates were used for each biological sample, and negative controls containing no template were used to exclude the possibility of DNA contamination. The same fluorescence threshold was set to identify the quantification cycle values for both genes, and the mean of the triplicate for each cDNA sample was used to determine ΔΔCt.

### Statistics

All statistical analyses were performed in IBM SPSS Statistics 24 (IBM SPSS Statistics for Windows, Version 24.0. Armonk, NY: IBM Corp.) and GraphPad Prism (GraphPad Prism version 7.00 for Windows, GraphPad Software, La Jolla California USA, www.graphpad.com). All comparisons were planned (i.e. prior to data collection), and accordingly, corrections for multiple comparisons were not required^99^. Scoring of the long-term odour recall test was binary and therefore analysed with logistic generalized estimating equations using the binomial error structure with the logit-link function, a recommended way to compare categorical data^100^. The dependent variable “response to only cage scent” was scored as “yes” if bees exhibited PER to the scent used in cages with scented sucrose and “no” if bees exhibited PER to both this and the novel scent or to no scents. The interaction term of “scent x electric shock” was included in analyses, and was removed from the model if it was not shown to be significant. Antennal lobe biogenic amine levels and sucrose responsiveness did not follow normal distributions; therefore, we used the non-parametric Mann Whitney U Test to compare groups, with missing data deleted pairwise (only specific missing values were excluded rather than entire cases, so as to include all available data). Octopamine receptor expression followed a normal distribution and displayed equal variances, so the parametric Student’s two-tailed *t*-test was used to compare expression levels between groups.

## Supplementary information

Dataset 1

## Data Availability

All data generated or analysed during this study are included in this published article (and its Supplementary Information Files).
